# Does periodontitis affect the treatment response of biologics in the treatment of rheumatoid arthritis?

**DOI:** 10.1186/s13075-020-02269-x

**Published:** 2020-07-25

**Authors:** Masahiro Tachibana, Yukio Yonemoto, Koichi Okamura, Takahito Suto, Hideo Sakane, Tetsuya Kaneko, Trang Thuy Dam, Chisa Okura, Tsuyoshi Tajika, Yoshito Tsushima, Hirotaka Chikuda

**Affiliations:** 1 Department of Orthopaedic Surgery, Gunma University Graduate School of Medicine, Showa-machi 3-39-15, Maebashi, Gunma 371-8511 Japan; 2 Department of Diagnostic Radiology and Nuclear Medicine, Gunma University Graduate School of Medicine, Showa-machi 3-39-15, Maebashi, Gunma 371-8511 Japan; 3 Radiology Center, Bach Mai Hospital, Hanoi, Vietnam; 4 Research Program for Diagnostic and Molecular Imaging, Division of Integrated Oncology Research, Gunma University Initiative for Advanced Research (GIAR), Maebashi, Gunma Japan

**Keywords:** Periodontitis, FDG-PET/CT, Biologic therapy, Rheumatoid arthritis, Treatment response

## Abstract

**Background:**

Rheumatoid arthritis (RA) and periodontitis (PD) have been suggested to share many clinical and pathological features. However, few reports have investigated the relationship between the degree of PD and the treatment response to RA.

This study aimed to examine the relationship between the extent of PD and the treatment response to biologics in RA patients using FDG-PET/CT.

**Methods:**

Sixty RA patients (male, *n* = 14; female, *n* = 46; average age, 58.3 years) treated with biologic agents were included in this study. FDG-PET/CT was performed at baseline and 6 months after the initiation of biological therapy. The maximum standardized uptake value (SUVmax) was used as a representative value for the assessment of the FDG uptake in periodontal tissue and joints including the bilateral shoulders, elbows, wrists, hip, knees, and ankle joints. The Disease Activity Score (DAS) 28-CRP and the following clinical parameters were assessed: C-reactive protein (CRP), erythrocyte sedimentation rate (ESR), anti-cyclic citrullinated peptide antibody (ACPA), rheumatoid factor (RF), and matrix metalloproteinase 3 (MMP-3). The relationship between the treatment response of RA and the baseline SUVmax of the periodontal tissue was evaluated.

**Results:**

The baseline periodontal SUVmax was related to patient age (*r* = 0.302, *p* = 0.009) and the ACPA level (*r* = 0.265, *p* = 0.025). The DAS28-CRP, CRP, ESR, MMP-3, and joint SUVmax values were significantly decreased after 6 months of biological therapy. However, the mean periodontal SUVmax, ACPA, and RF showed no significant changes after treatment. There was a significantly negative correlation between the baseline periodontal SUVmax and the treatment response of DAS28-CRP (*r* = − 0.369, *p* = 0.004).

**Conclusion:**

There was a negative correlation between the extent of PD at baseline and the treatment response of RA patients who received biological therapy. The evaluation of the periodontal condition is considered to be an essential part for the management of RA.

## Background

In recent years, rheumatoid arthritis (RA) and periodontitis (PD) have been suggested to share many clinical and pathological features and PD is considered to be a risk factor for RA. Some previous reports indicated the increased prevalence of PD in RA patients [1, 2]. PD causes persistent inflammation and is linked with citrullination. In RA patients, the severity of periodontal conditions was associated with both the presence and levels of anti-cyclic citrullinated peptide antibodies (ACPAs) [3]. Additionally, anti-cyclic citrullinated peptide antibody (ACPA) has been reported as an important indicator for RA treatment and is associated with erosive joint destruction [4, 5].

*Porthyomonas gingivalis* (*P. gingivalis*) is the main causative agent of the PD and a unique bacterium that is known to express an enzyme that increases citrullination [6, 7]. Recently, it has been reported that *P. gingivalis* can induce RA in non-smokers. PD associated with *P. gingivalis* is more likely to be present in those at risk of RA in comparison to healthy controls [8]. Furthermore, the presence of *P. gingivalis* infections may precede the clinical onset of RA by a number of years [9].

There has been no agreement on the relationship between the degree of PD and the disease activity of RA. Rodríguez-Lozano et al. reported that there was an association between the disease severity of PD and the disease activity of RA [10]. In contrast, Mobini et al. demonstrated that there was no significant relationship between the disease activity of RA and the severity of PD [11]. Additionally, it might be possible that PD affects the treatment response of RA. Savioli et al. reported that RA patients with PD showed no significant differences in disease activity parameters during 6 months of treatment with anti-tumor necrosis factor (TNF) inhibitors [12]. However, the extent of PD in their study was unknown, and the relationship between PD and the treatment response of RA has been unclear.

This study aimed to examine the relationship between the extent of PD and the treatment response in RA patients who receive biological therapy. We hypothesized that the degree of PD would be correlated with the treatment response in RA patients who receive biological therapy. To assess the degree of periodontitis, we used [18F] fluorodeoxyglucose-positron emission tomography/computed tomography (FDG-PET/CT) [13–16]. FDG-PET/CT has also been used to assess the disease activity of the RA [17–21].

## Materials and methods

### Patients and methods

The institutional review board of our hospital approved the study. Sixty patients (male, *n* = 14; female, *n* = 46; average age, 58.3 ± 14.1 years) were enrolled in this study (Table 1). Based on the power analysis, a sample size of 60 patients would supply 80.4% power to detect a difference with an alpha error of 0.05.

**Table 1:** The characteristics and the parameters of all patients at baseline and after treatment

*n* = 60	Baseline	After treatment	p*
Age (year)	58.3 ± 14.1	–	–
Sex (male/female)	14/46	–	–
Disease duration (year)	13.3 ± 12.4	–	–
Steinbrocker stage (I/II/III/IV)	7/14/21/18	–	–
Steinbrocker class (1/2/3/4)	14/27/13/6	–	–
Concomitant MTX (%)	55	–	–
MTX dosage (mg/week)	8.91 ± 2.65	–	–
Concomitant PSL (%)	45	–	–
PSL dosage (mg/day)	3.70 ± 1.86	–	–
Smoking history (%)	10	–	–
ACPA positive (%)	80	–	–
CRP (mg/dl)	2.07 ± 2.21	0.50 ± 1.05	< 0.01
ESR (mm/h)	62.6 ± 31.4	35.3 ± 27.0	< 0.01
WBC (/μl)	6610 ± 2140	5582 ± 2067	< 0.01
RF (mg/dl)	256.0 ± 876.9	162.7 ± 551.9	0.43
ACPA (U/ml)	91.5 ± 117.1	75.2 ± 96.1	0.36
MMP-3 (ng/ml)	261.30 ± 243.82	118.65 ± 129.42	< 0.01
DAS28-CRP	4.07 ± 1.23	2.35 ± 1.01	< 0.01
Joints SUV	2.13 ± 0.65	1.67 ± 0.50	< 0.01
Periodontal SUV	1.83 ± 0.46	1.88 ± 0.45	0.39

*Wilcoxon’s signed-rank test

Abbreviations: *MTX* methotrexate, *PSL* prednisolone, *ACPA* anti-cyclic citrullinated peptide antibody, *CRP* C-reactive protein, *ESR* erythrocyte sedimentation rate, *WBC* white blood cell, *RF* rheumatoid factor, *MMP-3* matrix metalloproteinase 3, *DAS* Disease Activity Score

All patients were diagnosed according to the American College of Rheumatology (ACR) criteria revised in 1987 [22], and their previous treatment with conventional synthetic disease-modifying antirheumatic drugs (csDMARDs), including methotrexate (MTX), provided clinically inadequate responses. Thus, the patients were recommended for treatment with biological agents. The following biological agents were administered: infliximab (IFX; *n* = 18), etanercept (ETN; *n* = 14), adalimumab (ADA; *n* = 15), and tocilizumab (TCZ; *n* = 13). Patients with poor control of diabetes and those with other inflammatory diseases were excluded from the analysis. The average disease duration of these patients was 13.3 ± 12.4 years. The concomitant MTX rate was 75%, the average dose of MTX was 6.99 ± 2.57 mg/week, the concomitant prednisolone (PSL) rate was 75%, and the average dose of PSL was 3.71 ± 1.86 mg/day. After a baseline assessment using whole-body FDG-PET/CT, they received biological therapies. FDG-PET/CT and clinical assessments were also performed 6 months after the initiation of therapy. The clinical assessments included measurement of the serum concentrations of C-reactive protein (CRP), erythrocyte sedimentation rate (ESR), matrix metalloproteinase 3 (MMP-3), ACPA, and rheumatoid factor (RF). The inflammation activity was evaluated using the Disease Activity Score (DAS) 28-CRP [23, 24].

### FDG-PET/CT image

Whole-body PET was performed following the intravenous injection of 18F-FDG (5 MBq/kg), after the patient had fasted for over 6 h. Data acquisition was performed in 3D mode, 60 min after the injection, using a PET-CT scanner (Biograph 16; Siemens Medical Solutions Inc., Munich, Germany). Patients were scanned from head to toe in the arms-down position, according to the methods of previous reports [17–21]. PET images were interpreted by physicians, and an increased FDG uptake in periodontal tissue and joints was recorded.

**Fig. 1:**
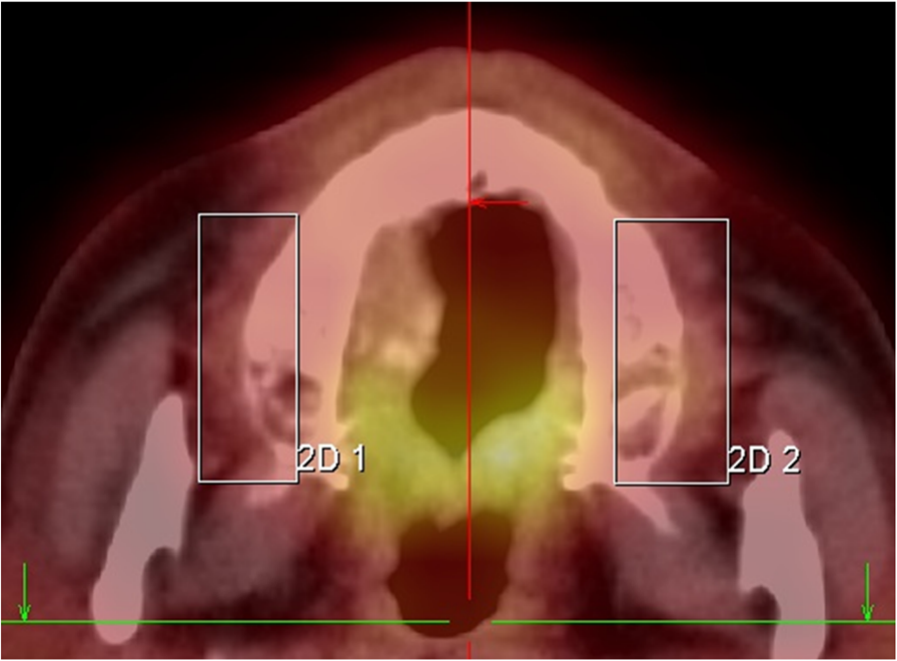
Periodontal measurement using FDG-PET/CT. FDG-PET/CT images demonstrate FDG activity localized to the periodontal tissues. The boxes within the PET-CT image indicate the region of FDG activity and are drawn from the middle of the teeth toward the ipsilateral aspect of the teeth. FDG-PET/CT, 18F-fluorodeoxyglucose-positron emission tomography, and computed tomography

To evaluate the FDG uptake in periodontal tissue, we measured the uptake in the upper posterior gingival tissue to limit spillover from pharyngeal and buccal structures [12–14] (Fig. 1).

To evaluate the FDG uptake in the joints, we measured 12 joints, including the bilateral shoulder, elbow, wrist, hip, knee, and ankle joints [17].

### Data analysis

For the semiquantitative analyses, functional images of the standardized uptake value (SUV) were produced using attenuation-corrected transaxial images, the injected doses of FDG, patient’s body weight, and the cross-calibration factor between PET and a dose calibrator. The SUV was defined as follows:

$$ \mathrm{SUV}=\mathrm{radioactive}\ \mathrm{concentration}\ \mathrm{in}\ \mathrm{the}\ \mathrm{region}\ \mathrm{of}\ \mathrm{in}\mathrm{terest}\ \left(\mathrm{ROI}\right)\left(\mathrm{MBq}/\mathrm{g}\right)/\mathrm{injected}\ \mathrm{dose}\left(\mathrm{MBq}\right)/\mathrm{patient}\hbox{'}\mathrm{s}\ \mathrm{body}\ \mathrm{weight} $$

ROIs were manually drawn on SUV images at each periodontal tissue and each joint including the bilateral shoulders, elbows, wrists, hip, knees, and ankle joints. The ROI analysis was conducted by a physician with the aid of corresponding CT scans. The maximal SUV (SUVmax) in the ROI was used as a representative value for the assessment of the FDG uptake.

The therapeutic response of the periodontal tissue and joints was evaluated by the changes in the mean SUVmax of the periodontal tissue and affected joints. The DAS28-CRP was also used to assess the treatment response. Periodontal SUV was defined as the mean SUVmax of the upper posterior gingival tissue. Joints SUV was defined as the mean SUVmax of the 12 joints.

In the following results section, Δ indicates the difference in the values before and after treatment.

For example, the ΔCRP was defined as follows:

$$ \Delta \mathrm{CRP}=\mathrm{CRP}\ \left(\mathrm{baseline}\right)-\mathrm{CRP}\ \left(\mathrm{after}\ \mathrm{treatment}\right) $$

### Statistical analysis

We performed a power analysis using GPower 3.1. A sample size of 60 patients would supply 80.4% power to detect a difference with an alpha error of 0.05 and an effect size of 0.354. Spearman’s rank correlation test and the partial correlation analyses were used to test the correlation between different parameters recorded in this study. Wilcoxon’s signed-rank test was used to assess differences in treatment evaluations. The IBM SPSS Statistics 24 software program (International Business Machines Corp., New York, NY, USA) was used for the analysis. *p* values of < 0.05 were considered to indicate statistical significance.

## Results

### Correlation between periodontal accumulation and RA parameters

The correlation between the baseline periodontal accumulation and RA parameters was examined. Periodontal accumulation was correlated with the level of ACPA (*r* = 0.354, *p* = 0.007). Age, RF, DAS28-CRP, and other parameters at baseline were not correlated with periodontal accumulation (Table 2).

**Table 2:** Relationship between the periodontal SUV and the clinical parameters at baseline

	*r*	p*
ACPA (U/ml)	0.354	0.01
Age (year)	0.206	0.11
RF (mg/dl)	0.073	0.59
DAS28-CRP	−0.228	0.08
Disease duration (year)	0.049	0.72
WBC (/μl)	−0.029	0.83
MMP-3 (ng/ml)	−0.107	0.43

*Spearman’s rank correlation test

Abbreviations: *ACPA* anti-cyclic citrullinated peptide antibody, *RF* rheumatoid factor, *DAS* Disease Activity Score, *WBC* white blood cell, *MMP-3* matrix metalloproteinase 3

### Changes before and after biological treatment

We examined the change of each parameter at baseline and 6 months after the initiation of the biological therapy (Table 1). After biological therapy, the CRP, ESR, MMP-3, joint SUVmax, and DAS28-CRP values were significantly decreased; however, the mean periodontal SUVmax was not significantly changed. Similarly, the ACPA and RF values showed no significant changes from before to after treatment.

We divided the patients into two groups, the anti-TNF-α inhibitor (IFX, ETN, ADA) treatment group and the anti-Interleukin-6 receptor inhibitor (TCZ) treatment group, and analyzed the groups in the same way. Neither group showed significant changes from before to after treatment (data not shown).

### Association between baseline parameters and the treatment response of RA patients who received biological therapy

There was a significant correlation between the periodontal SUVmax at baseline and ΔDAS28-CRP (*r* = − 0.369, *p* = 0.004) (Table 3). As for the citrullination, there was no correlation between the value of ACPA at baseline and changes in disease activity (data not shown). We used partial correlation coefficients to examine whether other baseline parameters had any effect on the correlations between the periodontal SUVmax at baseline and ΔDAS28-CRP. When the value of ACPA at baseline and the smoking history were taken as the control variable, there was no significant correlation between the periodontal SUVmax at baseline and ΔDAS28-CRP (*r* = − 0.213, *p* = 0.118 and *r* = − 0.246, *p* = 0.190, respectively) (Table 4).

**Table 3:** Relationship between the periodontal SUV at baseline and the difference in the values before and after treatment

	*r*	p*
ΔACPA (U/ml)	0.179	0.132
ΔWBC (/μl)	−0.063	0.634
ΔRF (mg/dl)	0.059	0.659
ΔMMP-3 (ng/ml)	−0.211	0.108
ΔDAS28-CRP	−0.369	0.004

*Spearman’s rank correlation test

Abbreviations: *ESR* erythrocyte sedimentation rate, *WBC* white blood cell, *RF* rheumatoid factor, *ACPA* anti-cyclic citrullinated peptide antibody, *MMP-3* matrix metalloproteinase 3, *DAS* Disease Activity Score, Δ the difference in the values between before and after treatment

**Table 4:** Partial correlations between the periodontal SUVmax at baseline and ΔDAS28-CRP

Control variables		ΔDAS28-CRP
Age (year)	*r*	−0.288
	*p*	0.027
Disease duration (year)	*r*	−2.777
	*p*	0.005
RF (mg/dl)	*r*	−0.27
	*p*	0.043
Smoking history	*r*	−0.246
	*p*	0.19
ACPA (U/ml)	*r*	−0.213
	*p*	0.118

*RF* rheumatoid factor, *ACPA* anti-cyclic citrullinated peptide antibody

## Discussion

Some previous studies reported that ACPA was higher in patients with severe PD in both RA and non-RA patients [6, 7]. The results of this study also showed a correlation between the periodontal accumulation of FDG and the level of ACPA (Table 2), suggesting that there was a relationship between periodontal disease and citrullination.

Regarding the use of FDG-PET/CT for the evaluation of periodontal disease, ^18^F-FDG is actually an excellent tracer for the detection of inflammation. Some human studies have demonstrated that useful information about the inflammation of a cavity can be obtained in addition to the detection of primary tumors, metastatic disease, and lymph node metastasis [13, 16, 24, 25]. The density of ^18^F-FDG is proportional to the degree of inflammation from oral infections [25].

A recent meta-analysis revealed the association between periodontitis and RA [26]. Rodríguez-Lozano et al. demonstrated that the severity of periodontitis was significantly associated with RA disease activity [10]. Because RA severity and the condition of PD are associated, therapy for one disease has potential to treat the other disease.

To date, the effect of biologics on PD has been investigated. A previous prospective study from France demonstrated that the periodontal parameters of 40 RA patients became worse with 33.9 months of IFX therapy [27]. Recently, Rinaudo-Gaujous et al. indicated that IFX therapy slightly increased concentrations of antibodies against *P. gingivalis* [28]. On the other hand, they recently reported that the periodontal parameters of RA patients (*n* = 21, average disease duration, 14 years) was improved after 6 months of anti-B lymphocyte therapy [29].

Marotte summarized these previous studies. TNF blocker treatment worsens the gingival inflammation, but decreases the gingival destruction of bone. In contrast, B cell blocker or IL-6 receptor blockers decreased the gingival inflammation or gingival bone destruction related to the PD [30–32]. In our study, there was no significant change in the periodontal accumulation of FDG from before to after biological treatment. Furthermore, there was no significant difference in the PD activity between the anti-TNF therapy group and the anti-IL-6 therapy group in our study. These data indicated that biological treatment, including anti-TNF and anti-IL6 receptor therapy, might not improve the periodontal disease activity of RA patients and that the difference in the mechanisms of biologic agents did not influence the disease activity of PD although further study should be undertaken to evaluate the effect of biologics on the condition of PD.

As for the treatment responses of RA patients with or without PD, a recent prospective study demonstrated that 6 months of treatment with an anti-TNFα inhibitor (IFX) did not improve the disease activity of RA in patients with PD; however, the disease activity of RA of the patients without PD was decreased by 6 months of IFX treatment [12]. Since they did not directly compare these two groups, and the extent of PD had not been investigated, the influence of PD on the treatment response of RA had not been understood. In our study, we evaluated the degree of periodontal inflammation according to the accumulation of FDG. The results of this study showed a negative correlation between the periodontal accumulation of FDG before biological treatment and the change of DAS28-CRP. These results indicated that the condition of periodontitis, which was associated with the ACPA levels, affected the treatment response of RA. It was considered that the poorer the PD state was, the less effective the biological treatment. Therefore, treatment intervention for PD might be worthwhile for improving the therapeutic response of patients with RA.

Another point is that the PD severity itself might affect the disease activity of RA. Some previous studies suggested that the treatment of PD could improve RA outcomes [33–35]. However, the ESPERA randomized controlled trial [36] recently concluded that periodontal treatment led to periodontal health, but no significant effects on the DAS28-ESR [37]. At any rate, good dental hygiene is important for RA patients.

Our study was associated with some limitations. First, periodontitis was evaluated based on the accumulation of FDG and the periodontal condition was not checked by a dentist. Second, we only evaluated patients with RA; there was no control group. Third, the FDG uptakes of the hand joints were not assessed in this study since the method used in this study was not suitable for measurement of the hands. With a special device, the hands should be scanned separately with the patient in the prone position.

## Conclusion

There was a negative correlation between the extent of PD at baseline and the treatment response of RA in patients who received biological therapy. Therefore, the condition of periodontitis might affect ACPA levels and the treatment response of RA. The evaluation of periodontal condition is considered an essential part for the management of RA.

## Data Availability

All data generated or analyzed during this study are included in this published article and its supplementary information file.

## Abbreviations

Δ: Differences in the values before and after treatment
ACPA: Anti-cyclic citrullinated peptide antibody
ACR: American College of Rheumatology
ADA: Adalimumab
CRP: C-reactive protein
csDMARDs: Conventional synthetic disease-modifying antirheumatic drugs
DAS: Disease activity score
ESR: Erythrocyte sedimentation rate
ETN: Etanercept
FDG-PET/CT: Fluorodeoxyglucose-positron emission tomography/computed tomography
IFX: Infliximab
MMP-3: Matrix metalloproteinase 3
MTX: Methotrexate
PD: Periodontitis
*P. gingivalis*: *Porthyomonas gingivalis*
PSL: Prednisolone
RA: Rheumatoid arthritis
RF: Rheumatoid factor
ROI: Region of interest
SUV: Standardized uptake value
SUVmax: Maximum standardized uptake value
TCZ: Tocilizumab
TNF: Tumor necrosis factor
